# Dihydroartemisinin inhibits activation of the AIM2 inflammasome pathway and NF-κB/HIF-1α/VEGF pathway by inducing autophagy in A431 human cutaneous squamous cell carcinoma cells

**DOI:** 10.7150/ijms.57167

**Published:** 2021-05-13

**Authors:** Yajie Wang, Zhijia Li, Muzhou Teng, Junlin Liu

**Affiliations:** 1 Department of Dermatology, Cosmetology and Venereology, Shenzhen Hospital, Southern Medical University, Shenzhen, 518101, Guangdong, China.; 2 Molecular Diagnosis and Treatment Center for Infectious Diseases, Dermatology Hospital of Southern Medical University, Guangzhou, 510091, Guangdong, China.; 3 Department of Dermatology, the Second Affiliated Hospital, Hainan Medical University, Haikou, 570311, Hainan, China.

**Keywords:** dihydroartemisinin, cutaneous squamous cell carcinoma, autophagy, AIM2 inflammasome pathway, NF-κB/HIF-1α/VEGF pathway

## Abstract

The therapeutic effect of dihydroartemisinin (DHA) against cutaneous squamous cell carcinoma (cSCC) has been previously demonstrated; however, the underlying mechanism remains unclear. This study sought to verify the therapeutic effect of DHA against cSCC and explore its underlying mechanism in A431 cSCC cells. This study reported that DHA inhibited A431 cells proliferation in a time- and concentration-dependent manner and promoted A431 cells apoptosis. Moreover, DHA inhibited the invasion and migration of A431 cells. Mechanistically, DHA promoted autophagy and inhibited activation of the absent in melanoma 2 (AIM2) inflammasome pathway and NF-κB/HIF-1α/VEGF pathway. Treatment of A431 cells with the mTOR inhibitor, and autophagy promoter, rapamycin also inhibited these two pathways. In conclusion, DHA inhibited activation of the AIM2 inflammasome pathway and NF-κB/HIF-1α/VEGF pathway by promoting autophagy in A431 cells, thus accounting for its therapeutic effect. Induction of autophagy by DHA may be mediated by inhibiting the mTOR pathway and promoting reactive oxygen species production.

## Introduction

Cutaneous squamous cell carcinoma (cSCC) is a malignant tumor originating from the epidermis or adnexal keratinocytes. The incidence of cSCC is increasing annually, causing it to become a global public health issue 1. The role of the inflammatory microenvironment in the development, invasion, and metastasis of cSCC has been widely recognized 2. Specifically, inflammasomes and nuclear factor-κB (NF-κB) reportedly participate in the inflammatory microenvironment, which affects cSCC 3-6.

Inflammasomes are important components of the innate immune response, which is involved in inflammation. As sensors of exogenous and endogenous danger signals, inflammasomes are triggered to secrete the proinflammatory cytokines, interleukin (IL)-1β and IL-18 7. Inflammasome complexes are composed of a cytoplasmic sensor, including NLRP1, NLRP3, and absent in melanoma 2 (AIM2), apoptosis-associated speck-like protein containing a caspase recruitment domain (ASC), and pro-caspase-1 8. However, the role of inflammasome activation in the occurrence and development of different tumors varies. One study reported that the expression of AIM2 in cSCC is significantly higher than that in normal skin tissues, while AIM2 knockdown reduces the viability of cSCC cells and initiates cell apoptosis, thus inhibiting the growth of cSCC xenografts 3. Furthermore, knockdown of AIM2 led to decreased expression of the proinvasive metalloproteinases MMP1 and MMP13, weakening the invasiveness of cSCC cells and inhibiting vascularization of cSCC xenografts 3. However, no studies have been performed that target inflammasomes for treating cSCC.

NF-κB is the molecular center connecting inflammation and cancer, and can regulate the behavior of tumor cells, as well as the inflammatory microenvironment in various ways 9. NF-κB has been shown to promote tumor cell proliferation, inhibit apoptosis, and stimulate cell migration and invasion 9, 10. Activation of NF-κB can up-regulate hypoxia-inducible factor-1α (HIF-1α) and vascular endothelial growth factor (VEGF), which leads to tumor vascularization 11, 12. The NF-κB/HIF-1α/VEGF pathway plays an important role in stimulating tumor cell survival, proliferation, invasion, and metastasis by inhibiting apoptosis and regulating the immune system 13, 14.

Autophagy, particularly macroautophagy, plays an important role in the intracellular balance by transferring cytoplasmic components to lysosomes for degradation and amino acid recycling 15. Autophagy inhibits the development of cancer via an autonomic antitumor function and by regulating inflammation and immunity 16. It has been confirmed that autophagy targets endogenous activators of inflammasomes to regulate inflammasome activation 15. Moreover, autophagy and the function of NF-κB interfere with each other 17.

Dihydroartemisinin (DHA) is a derivative of artemisinin and has been widely used as an antimalarial drug 18. Meanwhile, it has also been reported to exert therapeutic effects against cancers 19-24. DHA promotes cell apoptosis and inhibits cell proliferation by inducing autophagy in various carcinoma cells, including cervical carcinoma, cholangiocarcinoma, and hepatocellular carcinoma 19-21. However, whether DHA can inhibit the proliferation and promote the apoptosis of cSCC cells via autophagy remains unclear. Therefore, in this study, we investigated the effects of DHA on the proliferation, apoptosis, invasiveness, and migration ability of A431 cSCC cells and explored the mechanism underlying these effects.

## Methods

### Cell line and drug treatment

The A431 cSCC cell line was purchased from Boster Biological Technology Co., Ltd. (Wuhan, China) and cultured in Iscove's Modified Dulbecco's Medium (Boster Biological Technology) supplemented with 10% fetal bovine serum (HyClone, Logan, UT, USA), 100 U/mL penicillin, and 100 µg/mL streptomycin (HyClone) at 37 °C in humidified air containing 5% CO_2_. DHA was purchased from Meilunbio (Dalian, China). DHA, etoposide (Sigma, St. Louis, MO, USA), 3-methyladenine (3-MA; Sigma), and rapamycin (Sigma) were dissolved in dimethyl sulfoxide (DMSO; Sigma) and stored at -20 °C. A431 cells were treated with DHA (20 and 40 µM), etoposide (20 µM), or rapamycin (1 µM) for 24 h. Culture medium containing 0.1% DMSO was used as a control. For cotreatment with DHA, A431 cells were pretreated with 3-MA (5 µM) and rapamycin (10 µM) for 1 h.

### Cell viability assay

A431 cells were seeded into 96-well culture plates (1 × 10^4^ cells/well) and treated with various concentrations of DHA (0, 20, 40, and 60 µM) for 12, 24, and 48 h. Cell viability was evaluated using a Cell Counting Kit-8 (CCK-8; Dojindo Molecular Technologies, Kumamoto, Japan) according to the manufacturer's instructions. Absorbance was measured at 450 nm.

### Clone formation assay

A431 cells were treated with DHA (0, 20, or 40 µM) or etoposide (20 µM) for 24 h, digested with trypsin, and seeded into a 96-well plate at a density of 400 cells per well. After ten days of culture, cell colonies were fixed with anhydrous ethanol and stained with crystal purple. An AID EliSpot reader (Autoimmun Diagnostika, Strassberg, Germany) was used for analysis and scanning.

### Flow cytometry

The A431 cells were treated with DHA (0, 20, or 40 and 60 µM) for 24 hours, digested with trypsin, washed with PBS, and suspended with binding buffer. Then, the cells were stained with Annexin V-FITC and PI reagent (Dojindo Molecular Technologies) in the dark for 15 minutes, according to the manufacturer's instructions. The degree of apoptosis was measured by flow cytometry analysis.

### Transwell invasion assay

The invasiveness of A431 cells was determined using a Transwell invasion assay. Matrigel was diluted 1:3 in an FBS-free medium and spread onto the upper surface of a Transwell insert at a density of 1 × 10^5^ cells/well. The lower chamber contained 600 µL of Iscove's Modified Dulbecco's Medium supplemented with 10% FBS. The DHA and negative control (NC) groups were treated with 40 µM and 0 µM DHA, respectively, for 24 h. Invaded cells on the bottom polycarbonate membrane were fixed with 4% paraformaldehyde and stained with crystal purple. Images of invaded cells were obtained under an inverted microscope.

### Wound healing assay

A431 cells (1 × 10^6^ cells/mL) were seeded into 6-well culture plates. After the cell layer was scratched with a sterile micropipette tip, the cells were treated with DHA (0 µM and 40 µM) in serum-free medium. The cell layers were photographed at 0, 24, 48, and 72 h after scratching, and wound healing was observed under an inverted microscope.

### Determination of reactive oxygen species (ROS) level

A431 cells were cultured in 6-well plates and stimulated with 0, 20, and 40 µM DHA for 24 h in the NC group, 20 µM DHA group, and 40 µM DHA group, respectively. After treatment, the liquid in the plates was discarded. Next, 300 µL DCFH-DA (10 mmol/L) was added and the cells were cultured at 37 °C in the dark for 20 min. DCFH-DA was provided with the ROS detection kit (Beyotime Biotechnology, Shanghai, China). The plates were shaken every 3-5 min to ensure mixing of the reagent and cells. After incubation, the liquid was discarded, and the plates were washed with PBS three times to remove excess DCFH-DA. Images were acquired using a fluorescence microscope (DMI6000B; Leica, Wetzlar, Germany).

### Real-time fluorescence-based quantitative PCR

Real-time fluorescence-based quantitative PCR was performed to determine the LC3B, AIM2, IL-1β, and IL-18 mRNA expression levels. Total RNA was extracted from A431 cells after cell fragmentation and lysis. cDNA was synthesized using a reverse transcription kit under the following reaction conditions: 42 °C for 15 min and 85 °C for 5 min, followed by cooling to 4 °C. qPCR amplification was performed with cDNA from each group on a Rotor-Gene 6000 fluorescence quantitative PCR instrument (Corbett Research, Sydney, Australia). The amplification conditions were as follows: 96 °C for 30 s, followed by 40 cycles at 94 °C for 5 s, 60 °C for 30 s, and 72 °C for 10 s. After amplification, the Ct values, and ΔΔCt values were obtained.

### Western blot analysis

RIPA lysis buffer (Solarbio, Beijing, China) containing phosphotransferase inhibitors and PMSF was used to extract proteins. Protein concentrations were determined using a BCA protein assay kit (Boster Biological Technology). Proteins were separated by SDS-PAGE and electroblotted onto polyvinylidene fluoride membranes, which were blocked with 5% nonfat milk and incubated with primary antibodies at 4 °C overnight. The membranes were then incubated with secondary antibodies, and protein bands were visualized using an electrochemiluminescence detection system. The antibodies used in this study were rabbit anti-AIM2 (1:1,000; ab93015, Abcam, Cambridge, UK), rabbit anti-IL-1β (1:1,000; 16806-1-AP, Proteintech, Rosemont, IL, USA), rabbit anti-IL-18 (1:1,000; ab191152, Abcam), rabbit anti-LC3B (1:1,000; #2775, Cell Signaling Technology, Danvers, MA, USA), rabbit anti-NF-κB (1:1,000; ab207297, Abcam), rabbit anti-HIF-1α (1:1,000; ab179483, Abcam), rabbit anti-VEGF (1:1,000; sc-152, Santa Cruz Biotechnology, Santa Cruz, CA, USA), rabbit anti-p-mTOR (Ser2448) (1:1,000; #5536, Cell Signaling Technology), rabbit anti-mTOR (1:1,000; #2983, Cell Signaling Technology), rabbit anti-p-p70S6K (Thr389) (1:1,000; #97596, Cell Signaling Technology), rabbit anti-p70S6K (1:1,000; #2708, Cell Signaling Technology), horseradish peroxidase-conjugated monoclonal mouse anti-GAPDH (1:10,000; KC-5G5, Aksomics, Shanghai, China), and goat anti-rabbit horseradish peroxidase-conjugated IgG (1:5,000; 4050-05, Southern Biotech, Birmingham, AL, USA).

### Immunofluorescence assay

A431 cells were cultured on glass coverslips in a 24-well plate (2 × 10^5^ cells/well) for 24 h. The cells were fixed, permeabilized, blocked, and incubated with a primary antibody at 4 °C overnight, followed by incubation with a secondary antibody at 37 °C for 1 h. Rabbit anti-LC3B (1:10,000; #43566, Cell Signaling Technology) was used as a primary antibody, and Alexa Fluor® 488-conjugated anti-rabbit IgG (H+L) (1:500; #4412, Cell Signaling Technology) was used as a secondary antibody. The cytoskeleton was stained with phalloidin (Sigma) for 1 h, and the nuclei were stained with DAPI (Sigma) for 10 min. Images were acquired using a fluorescence microscope (DMI6000B; Leica).

### Small interference RNA (siRNA) transfection

ATG5 siRNAs (GenePharma, Shanghai, China) were transfected into A431 cells to genetically inhibit autophagy flux. Briefly, ATG5 siRNAs/Control siRNAs and Lipofectamine 2000 (Invitrogen, Calsbad, CA, USA) were diluted with Opti-MEM (Invitrogen), followed by incubation for 5 min. Then, the diluted Lipofectamine 2000 and siRNAs were mixed gently and incubated for 20 min at room temperature to form complexes. Next, the siRNAs/Lipofectamine 2000 complexes were added to each well containing the A431 cells and medium. The A431 cells were incubated at 37 °C in a CO_2_ incubator for 48 hours, and the transfection efficiency was evaluated using western blot analysis.

### Statistical analysis

All experiments were repeated a minimum of three times, and the results were presented as mean ± SD. Student's *t*-test was used to determine the significance of differences. A value of *p* < 0.05 was considered statistically significant.

## Results

### DHA inhibits the proliferation and induces the apoptosis of A431 cells

**Figure 1:**
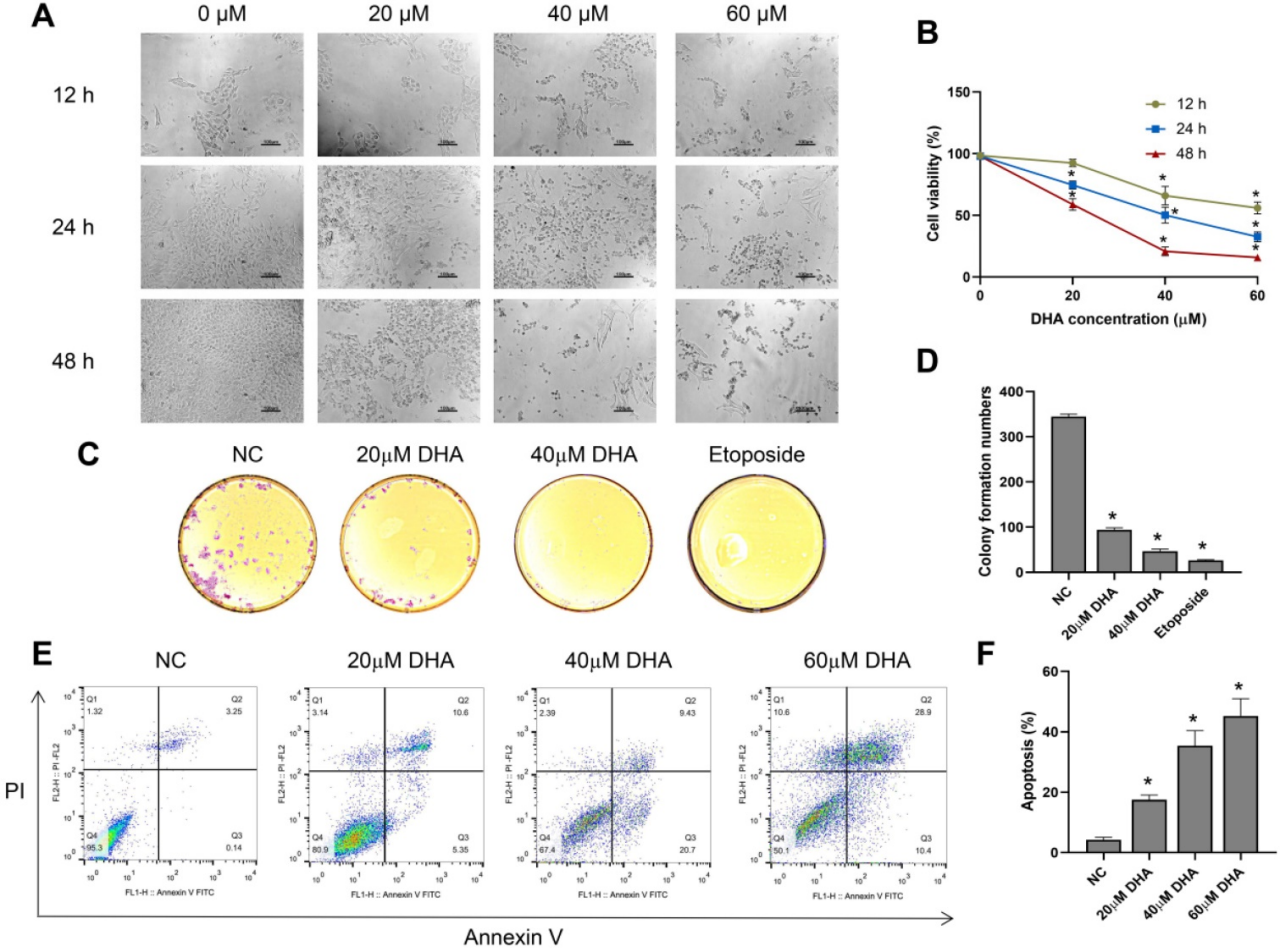
Effects of DHA on the proliferation and apoptosis of A431 cells. (a) Phase-contrast microscopy images of A431 cells treated with different concentrations of DHA for the indicated periods of time (100×). Scale bars = 100 µm. (b) Viability of A431 cells treated with different concentrations of DHA (mean ± SD, *n* = 3). *p < 0.05 versus untreated control. (c) Representative photographs of colony formation by untreated (NC) A431 cells and cells treated with DHA (20 or 40 µM) or 20 µM etoposide (positive control) for 24 h. (d) Quantification of colony formation in the above groups (mean ± SD, *n* = 3). *p < 0.05 versus NC group. (e) A431 cells were treated with DHA (0, 20, 40 and 60 µM) for 24 hours, and the degree of apoptosis was evaluated via flow cytometry analysis. (f) Quantification of apoptosis rates of cells from the above groups (mean ± SD, *n* = 3). *p < 0.05 versus NC group.

To investigate the effect of DHA on the growth and morphology of A431 cells, we treated the cells with different concentrations of DHA (0, 20, 40, and 60 µM) for 12, 24, and 48 h. With increasing DHA concentrations and treatment times, the cell number decreased, and cell state worsened (Figure 1A). Subsequently, we measured cell viability in each treatment group with a CCK-8 assay. The results showed that DHA inhibited the proliferation of A431 cells in a time- and concentration-dependent manner (Figure 1B) with 50% inhibitory concentrations of approximately 40 and 25 µM at 24 and 48 h, respectively.

Next, we evaluated the effect of DHA (20 and 40 µM) on colony formation by A431 cells using a clone formation assay, with etoposide (20 µM) used as a positive control. The results showed that colony formation by A431 cells treated with DHA for 24 h 25 significantly decreased, and the effect was similar to that of etoposide (Figure 1C, 1D).

Furthermore, we explored the effects of DHA (20, 40 and 60 µM) on the apoptosis of A431 cells by flow cytometry analysis. The results showed that the apoptosis rate of A431 cells increased markedly after treatment with DHA for 24 h (Figure 1E, 1F). Taken together, these findings indicate that DHA inhibits the proliferation and induces the apoptosis of A431 cells.

### DHA inhibits invasion and migration of A431 cells

**Figure 2:**
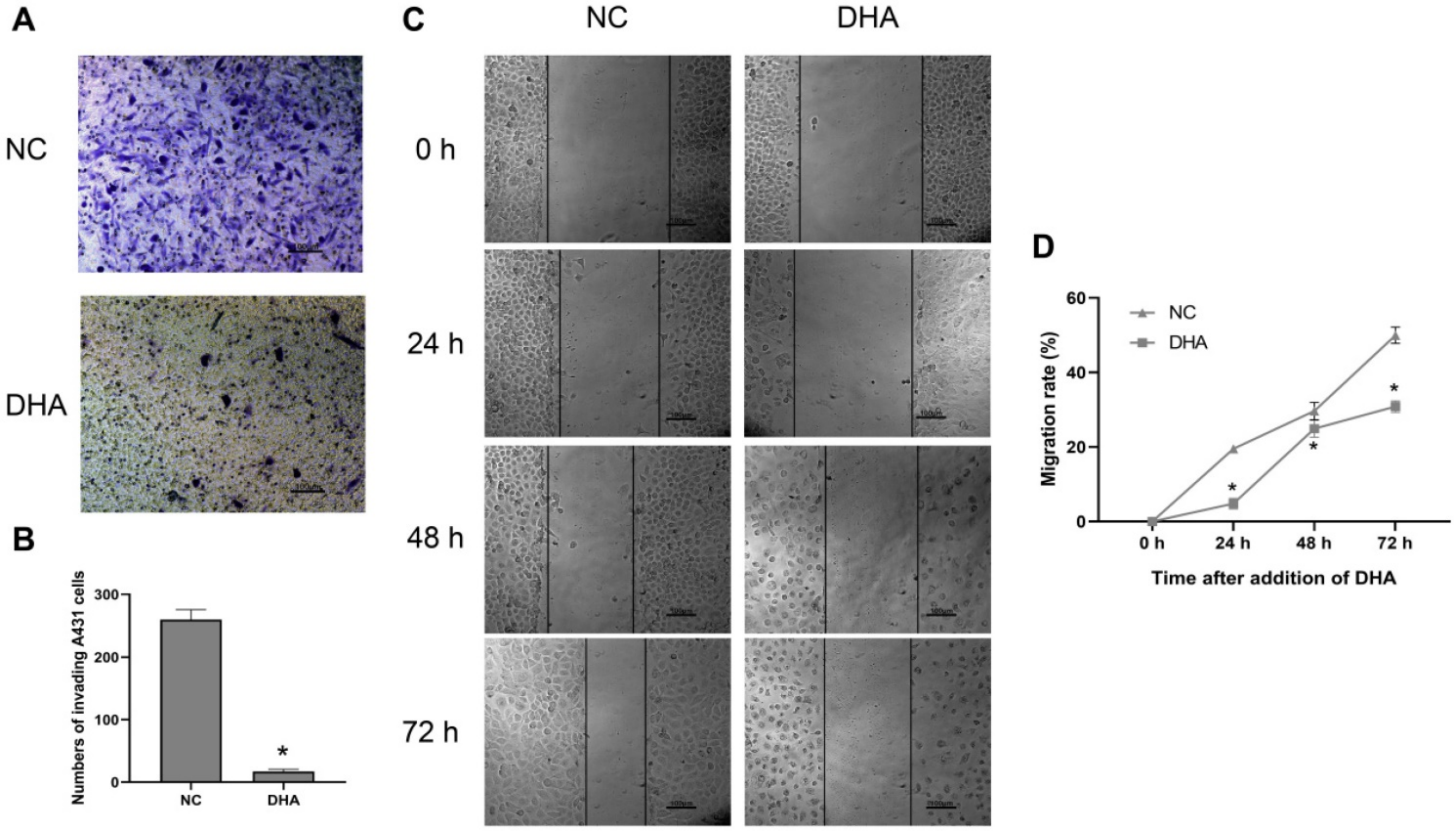
Effects of DHA on invasion and migration of A431 cells. (a) Representative images of cell invasion in the NC and DHA groups, treated with 0 and 40 µM DHA, respectively, for 24 h. Scale bars = 100 µm. (b) Quantification of invaded A431 cells in the NC and DHA groups (mean ± SD, *n* =3). *p < 0.05 versus NC group. (c) Wound healing in the NC and DHA (40 µM) groups at 24, 48, and 72 h. Scale bars = 100 µm. (d) Quantitative analysis of cell migration rates in the NC and DHA groups at 24, 48, and 72 h (mean ± SD, *n* = 3). Migration rate (%) = (A0 - An)/A0 × 100, where A0 represents the initial wound width and An represents the wound width at the metering point. *p < 0.05 versus NC group.

Clinically, the invasion and metastasis of cSCC often lead to a poor prognosis 26. The invasion ability of A431 cells was evaluated after treating the cells with 40 µM DHA for 24 h (Figure 2A, B). Microscopic observation showed that the number of A431 cells that invaded through the Matrigel was significantly lower in the DHA group than in the NC group. These results suggest that DHA inhibited the invasive ability of A431 cells. Furthermore, the effect of DHA on the migration ability of A431 cells was examined in a wound healing assay (Figure 2C, 2D). The results showed that the migration rate of A431 cells treated with 40 µM DHA for 24, 48, and 72 h was significantly lower than that in the NC group, suggesting that DHA significantly inhibited the migration ability of A431 cells.

### DHA induces autophagy in A431 cells

**Figure 3:**
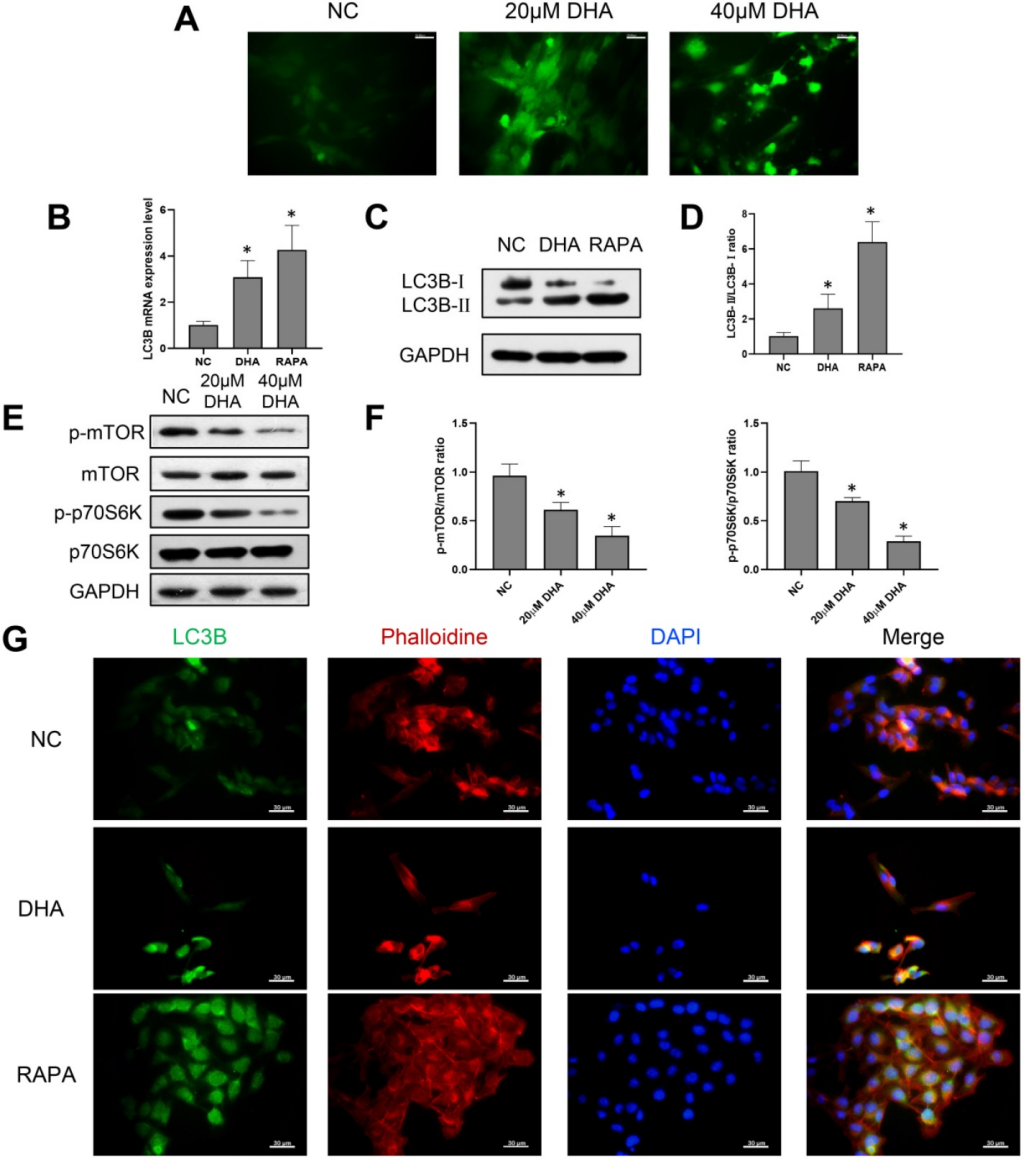
Induction of autophagy by DHA in A431 cells. (a) Representative images of ROS generation in the NC, 20 µM DHA, and 40 µM DHA groups, treated with 0, 20 and 40 µM DHA, respectively, for 24 h (400×). Scale bars = 30 µm. (b) mRNA expression levels of LC3B in untreated (NC) A431 cells and cells treated with 40 µM DHA or 1 µM rapamycin (autophagy positive control) for 24 h. GAPDH served as the endogenous control. Results are expressed as the mean ± SD of three independent experiments. *p < 0.05 versus untreated control. (c) Expression levels of the LC3B protein in the NC, DHA, and rapamycin groups. (d) LC3B-II/LC3B-I ratios in the NC, DHA, and rapamycin groups (mean ± SD, *n* = 3). *p < 0.05 versus NC group. (e) Expression levels of the mTOR, p-mTOR, p70S6K, and p-p70S6K proteins in the NC, 20 µM DHA, and 40 µM DHA groups. (f) p-mTOR/mTOR ratios and p-p70S6K/p70S6K ratios in the NC, 20 µM DHA, and 40 µM DHA groups (mean ± SD, *n* = 3). *p < 0.05 versus NC group. (g) Immunofluorescence analysis of autophagosome formation in A431 cells treated as described above (400×). Autophagosomes were labeled with an anti-LC3B antibody (green); cytoskeleton (F-actin) was stained with phalloidin (red), and nuclei were stained with DAPI (blue). Scale bars = 30 µm.

Considering that the production of ROS can mediate autophagy, A431 cells were treated with 0, 20, and 40 µM DHA for 24 h, and the production of ROS after DHA treatment was detected 27. The green fluorescence intensity represented the amount of ROS (Figure 3A). The results showed that the green fluorescence intensity of 20 and 40 µM DHA groups was significantly enhanced compared with the NC group. This suggests that the production of ROS increased after DHA treatment; that is, A431 cells experienced oxidative stress after DHA treatment. To explore the effect of DHA on autophagy, A431 cells were treated with 40 µM DHA and 1 µM rapamycin as a positive control for 24 h, after which the mRNA and protein expression of the autophagy-related marker, LC3B, was determined by real-time quantitative PCR and western blotting, respectively (Figure 3B-D). LC3B is currently the most widely used indicator of autophagic activity 28. Autophagosome formation is associated with the conversion of LC3B-I to LC3B-II, and the level of LC3B-II is closely correlated with the number of autophagosomes. Rapamycin induces autophagy by downregulating the activity of mTORC1, which inhibits autophagy. The results suggest that DHA promotes autophagy in A431 cells with effects similar to that of rapamycin.

Furthermore, inhibition of the mammalian target of rapamycin (mTOR) pathway can promote autophagy. Thus, the relative expression levels of proteins involved in the mTOR pathway in A431 cells were analyzed (Figure 3E, 3F). The ribosomal p70S6 kinase protein (p70S6K) is an important downstream effector of mTOR 29. The results showed that DHA significantly inhibited the expression of p-mTOR and p-p70S6K, causing mTOR pathway inhibition in A431 cells. Therefore, DHA may promote autophagy by inhibiting the mTOR pathway.

Further, LC3B was labeled by immunofluorescence to evaluate the presence of autophagosomes in A431 cells. As shown in Figure 3G, autophagosomes appeared as green dots under a fluorescence microscope, and DHA promoted their formation in A431 cells. Thus, autophagy activation in A431 cells may be associated with the anti-cSCC effect of DHA.

### DHA inhibits activation of the AIM2 inflammasome pathway by promoting autophagy

**Figure 4:**
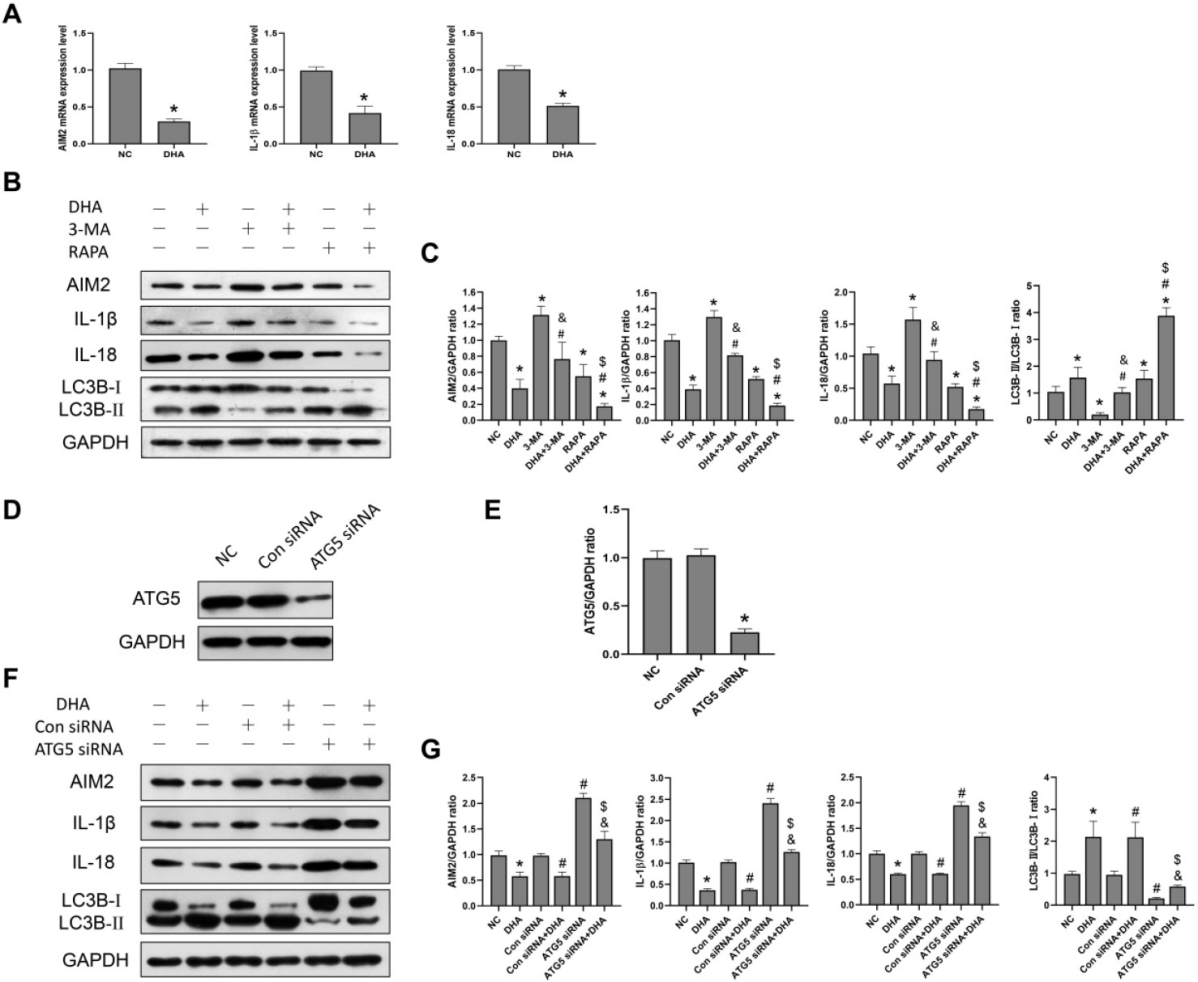
Inhibition of activation of the AIM2 inflammasome pathway by DHA via promoting autophagy. (a) mRNA expression levels of AIM2, IL-1β, and IL-18 in untreated (NC) A431 cells and cells treated with 40 µM DHA for 24 h (mean ± SD, *n* = 3). GAPDH served as the endogenous control. *p < 0.05 versus untreated control. (b) Expression levels of the LC3B, AIM2, IL-1β, and IL-18 proteins in untreated (NC) A431 cells and cells treated with DHA, 3-MA, 3-MA + DHA, RAPA, and RAPA + DHA. The 3-MA and 3-MA + DHA groups were pretreated with 5 µM 3-MA for 1 h, and the RAPA and RAPA + DHA groups were pretreated with 10 µM rapamycin for 1 h, followed by treatment of the DHA, 3-MA + DHA, and RAPA + DHA groups with 40 µM DHA for 24 h. (c) Quantitative analysis of the LC3B-II/LC3B-I, AIM2, IL-1β, and IL-18 levels in the above groups (mean ± SD, *n* = 3). *p < 0.05 versus NC group; ^#^*p* < 0.05 versus DHA group; ^&^*p* < 0.05 versus 3-MA group; ^$^*p* < 0.05 versus RAPA group. (d) Expression levels of the ATG5 protein in untreated (NC) A431 cells and cells treated with Control siRNA (Con siRNA) and ATG5 siRNA. (e) ATG5/GAPDH ratios in the NC, Con siRNA, and ATG5 siRNA groups (mean ± SD, n = 3). *p < 0.05 versus NC group. (f) Expression levels of the LC3B, AIM2, IL-1β, and IL-18 proteins in untreated (NC) A431 cells and cells treated with DHA, Control siRNA, Control siRNA + DHA, ATG5 siRNA, and ATG5 siRNA + DHA. A431 cells were transfected with Control siRNA or ATG5 siRNA for 48 h, and were treated with 40 µM DHA for another 24 h. (g) Quantitative analysis of the LC3B-II/LC3B-I, AIM2, IL-1β, and IL-18 levels in the above groups (mean ± SD, *n* = 3). There were no significant differences between the NC and Con siRNA groups and between the DHA and Con siRNA + DHA groups. *p < 0.05 versus NC group; ^#^*p* < 0.05 versus Con siRNA group; ^&^*p* < 0.05 versus Con siRNA + DHA group; ^$^*p* < 0.05 versus ATG5 siRNA group.

AIM2 is a sensor of cytoplasmic double-stranded DNA, and plays an important role in inflammasome activation 30. Activation of an AIM2 inflammasome can promote the production of the proinflammatory cytokines, IL-1β and IL-18, as well as the process of pyroptosis 30. Previously, the expression of AIM2 was reported to be significantly higher in cSCC compared to normal skin tissues, while its knockdown led to decreased cSCC cell viability 30. In the current study, after A431 cells were treated with 40 µM DHA for 24 h, the mRNA and protein expression levels of AIM2, IL-1β, and IL-18 were significantly lower than those in the NC group (Figure 4A, 4B), suggesting that DHA inhibits activation of the AIM2 inflammasome pathway in A431 cells.

To further explore the relationship between autophagy and the AIM2 inflammasome pathway, we pretreated A431 cells with the autophagy inhibitor, 3-MA, and autophagy activator, rapamycin, before treatment with DHA. As shown in Figure 3B, the expression of AIM2, IL-1β, and IL-18 was significantly higher in the 3-MA + DHA group and significantly lower in the RAPA + DHA group compared to the DHA-only group. Treatment of A431 cells with 3-MA or rapamycin alone activated or inhibited the AIM2 inflammasome pathway, respectively. These results suggest that DHA inhibits activation of the AIM2 inflammasome pathway by promoting autophagy in A431 cells.

In addition, we also knocked out the autophagy-related gene ATG5 to inhibit autophagy in A431 cells treated with ATG5 siRNA to reconfirm the relationship between autophagy and the AIM2 inflammasome pathway (Figure 4D, 4F). ATG5 plays the role of elongating and enwrapping cytosolic cargos and promoting the formation of autophagosomes 31. As shown in Figure 4F, the expression of AIM2, IL-1β, and IL-18 in the ATG5 siRNA+DHA group was significantly higher than that in the Control siRNA+DHA group. Treatment of A431 cells with ATG5 siRNA alone activated the AIM2 inflammasome pathway. These results demonstrate that DHA inhibits activation of the AIM2 inflammasome pathway in A431 cells by promoting autophagy.

### DHA inhibits activation of the NF-κB/HIF-1α/VEGF pathway by promoting autophagy

**Figure 5:**
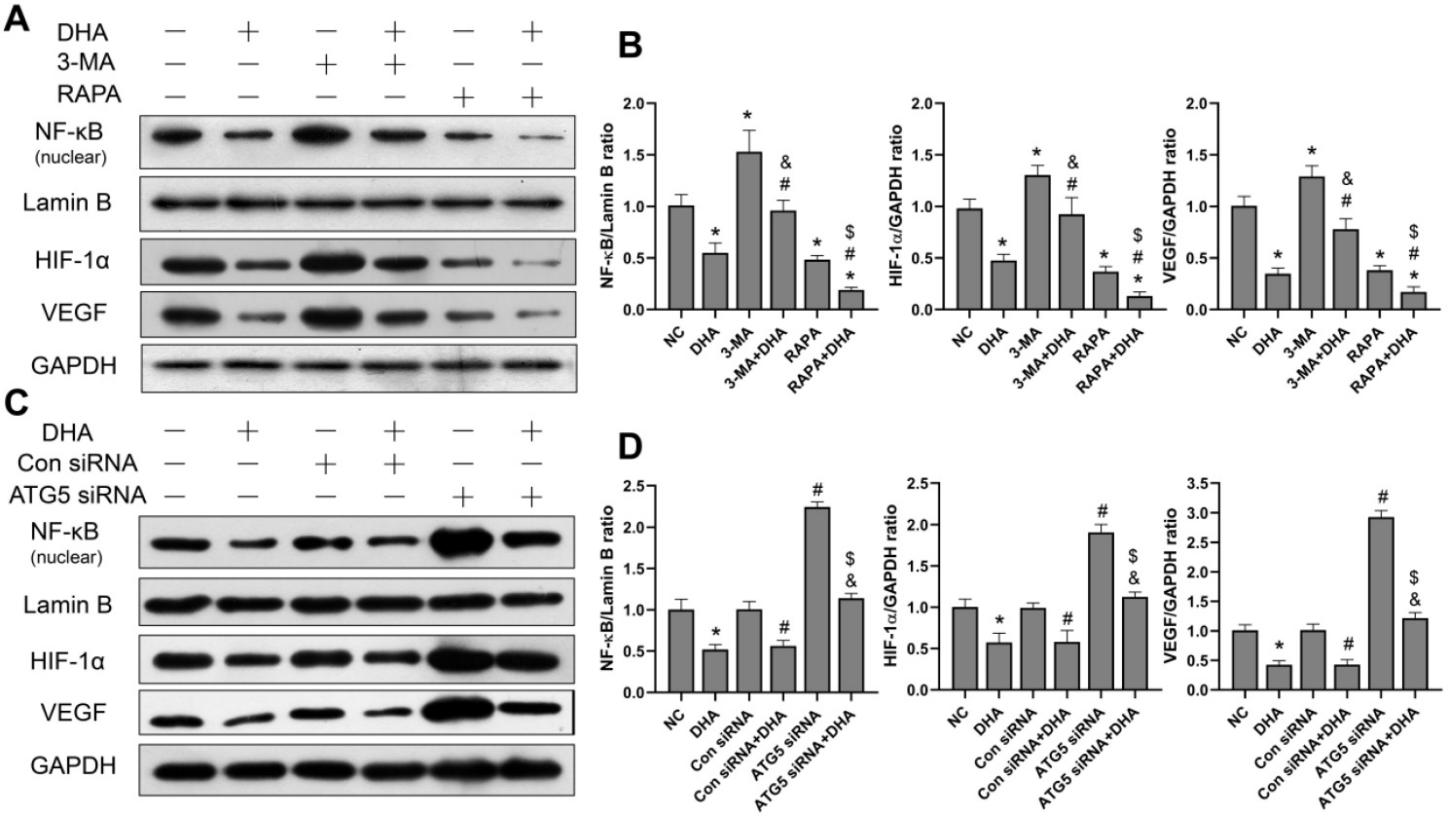
Inhibition of activation of the NF-κB/HIF-1α/VEGF pathway by DHA via promoting autophagy. (a) Expression levels of the NF-κB, HIF-1α and VEGF proteins in untreated (NC) A431 cells and cells treated with DHA, 3-MA, 3-MA + DHA, RAPA, and RAPA + DHA. (c) Quantitative analysis of the NF-κB, HIF-1α and VEGF levels in the above groups (mean ± SD, n = 3). *p < 0.05 versus NC group; ^#^*p* < 0.05 versus DHA group; ^&^*p* < 0.05 versus 3-MA group; ^$^*p* < 0.05 versus RAPA group. (c) Expression levels of the NF-κB, HIF-1α, and VEGF proteins in untreated (NC) A431 cells and cells treated with DHA, Control siRNA, Control siRNA + DHA, ATG5 siRNA, and ATG5 siRNA + DHA. (d) Quantitative analysis of the NF-κB, HIF-1α, and VEGF levels in the above groups (mean ± SD, n = 3). There were no significant differences between the NC and Con siRNA groups and between the DHA and Con siRNA + DHA groups. *p < 0.05 versus NC group; ^#^*p* < 0.05 versus Con siRNA group; ^&^*p* < 0.05 versus Con siRNA + DHA group; ^$^*p* < 0.05 versus ATG5 siRNA group.

To investigate the effect of DHA on the NF-κB/HIF-1α/VEGF pathway, as well as the relationship between DHA-induced autophagy and the pathway, the protein expression levels of NF-κB, HIF-1α, and VEGF in A431 cells were measured. The grouping and processing are the same as in section 3.4 (Figure 5). After activation, NF-κB enters the nucleus, where it regulates and encodes a variety of cytokines 32. The level of NF-κB protein in the nucleus of A431 cells was, therefore, detected. Results show that the expression of NF-κB, HIF-1α, and VEGF in the DHA group was significantly lower than that in the NC group, suggesting that DHA inhibits activation of the NF-κB/HIF-1α/VEGF pathway in A431 cells (Figure 5A, 5B). Further, expression of NF-κB, HIF-1α, and VEGF was significantly higher in the 3-MA + DHA group, and significantly lower in the RAPA + DHA group compared to the DHA-alone group. Treatment of A431 cells with 3-MA or rapamycin alone activated or inhibited the NF-κB/HIF-1α/VEGF pathway, respectively (Figure 5A, 5B).

Similarly, as shown in Figure 5C, the expression of NF- κB, HIF-1α, and VEGF in the ATG5 siRNA+DHA group was significantly higher than that in the Control siRNA+DHA group. Treatment of A431 cells with ATG5 siRNA alone activated the NF-κB/HIF-1α/VEGF pathway. Taken together, these results suggest that DHA inhibits the activation of the NF-κB/HIF-1α/VEGF pathway in A431 cells by promoting autophagy.

## Discussion

cSCC is a type of skin cancer that originates from keratinocytes and has strong metastatic potential 33. Age, race, exposure to ultraviolet radiation, sun-sensitive skin, and immunosuppression are classic risk factors for cSCC 33, 34. In most cases, nonmetastatic cSCC can be cured by surgical resection. However, new therapeutic strategies and drugs are still needed for metastatic, recurrent, and unresectable cSCC cases. DHA, a derivative of artemisinin, has a high antimalarial efficacy and is, thus, an important drug for treating malaria 18. In recent years, the anticancer effects of DHA have also been confirmed in a variety of tumor models. With regard to skin cancer, the inhibitory effects of DHA on the growth of cSCC and melanoma cells have been preliminarily confirmed 22-24. Its anticancer mechanisms have been shown to be diverse, including the promotion of apoptosis, activation of endoplasmic reticulum stress, acceleration of Fe^2+^-induced oxidative damage, and promotion of autophagy in cancer cells 21, 35, 36. In this study, we evaluated the therapeutic effect of DHA against cSCC and explored the underlying mechanism. We found that DHA inhibits the proliferation of A431 cSCC cells in a time- and concentration-dependent manner, and the inhibitory effect of DHA on clone formation by A431 cells was similar to that of etoposide. In addition, DHA promotes the apoptosis of A431 cells. Our results are consistent with those of a previous study by Hui et al, who found that DHA inhibits the proliferation and induces the apoptosis of A431 cells by inhibiting the Wnt/β-catenin pathway, but has little effect on the proliferation and apoptosis of normal human HaCaT keratinocytes 22. Invasive and metastatic cSCC is often associated with poor prognosis and a risk of death. Our results showed that DHA significantly inhibits the invasion and migration of A431 cells.

Autophagy and cell proliferation are two important processes in the pathophysiology of tumorigenesis 37. Excessive proliferation leads to accumulation of malignant tumor cells, whereas insufficient autophagy results in reduced capacity to eliminate mutant or damaged organelles in the process of malignant transformation 38. Importantly, autophagy can inhibit the development of cancer through its cell-autonomous antitumorigenic functions, as well as via regulation of inflammation and immunity 16, 39, 40. Compared to that in normal skin tissues and HaCaT cells, the conversion ratio of the autophagy-related protein LC3-I to LC3-II is significantly decreased in cSCC tissues and A431 cells, and autophagy flux is suppressed 41. DHA has been shown to promote apoptosis, or inhibit proliferation, of certain cancer cells by inducing autophagy, including cervical cancer, cholangiocarcinoma, and hepatocellular carcinoma 19-21. To further explore the therapeutic mechanism of DHA in cSCC, we verified the effect of DHA on autophagy in A431 cells. Results show that DHA promotes autophagy in A431 cells with effects similar to that of rapamycin. Moreover, close associations have been described between ROS and autophagy with ROS-induced oxidative stress causing activation of autophagy 27. In this study, we demonstrated that DHA promotes ROS production and activates oxidative stress in A431 cells. Inhibition of the mTOR pathway has also been shown to promote autophagy; however, we demonstrated that DHA significantly inhibits the expression of p-mTOR and p-p70S6K in A431 cells, thereby serving as an mTOR pathway inhibitor in A431 cells. Hence, DHA may elicit its therapeutic effects by inducing autophagy in A431 cells, which may be mediated by the inhibition of the mTOR pathway and promotion of ROS production.

Chronic inflammation promotes the occurrence and development of cSCC, in which inflammasomes are an important part of the inflammatory microenvironment. As an innate immunosensor, the AIM2 inflammasome specifically detects DNA in the cytoplasm, including damaged or abnormal endogenous DNA, as well as exogenous DNA of bacteria, viruses, and parasites 44. When dsDNA binds to AIM2, the AIM2 inflammasome pathway is activated to process pro-IL-1β and pro-IL-18 into the active proinflammatory cytokines IL-1β and IL-18, respectively, and to induce pyroptosis 45-47. IL-1β and IL-18 can promote carcinogenesis and tumor progression by inducing inflammation. Moreover, stimulation of a skin model with IL-1β and IL-18 was shown to induce epidermal hyperplasia and promote tumor formation 48, 49. Previous studies have also demonstrated that AIM2 expression is significantly upregulated in cSCC and its expression is positively correlated with the degree of cSCC malignancy 48, 49. Meanwhile, inhibition of AIM2 expression can inhibit the proliferation, and promote apoptosis, of cSCC cells, thus inhibiting the growth of a transplanted cSCC tumor 3. AIM2 knockout in cSCC cells led to decreased expression of invasive proteases MMP1 and MMP13, thereby weakening the invasion of tumor cells 3. Based on the results of this previous study, we predicted that DHA plays therapeutic role in cSCC by affecting the AIM2 inflammasome pathway. Indeed, we showed that DHA inhibits the expression of AIM2, IL-1β, and IL-18 in A431 cells, confirming that DHA inhibits the activation of the AIM2 inflammasome pathway in these cells. Based on the data from this, and previous studies, we postulate that DHA may effectively treat cSCC by inhibiting activation of the AIM2 inflammasome pathway.

NF-κB is the molecular hub that connects inflammation and cancer and participates in regulating the inflammatory response in the tumor microenvironment 9. After activation, NF-κB enters the nucleus and regulates the expression of many proteins, including cytokines, growth factors and cell adhesion molecules, thus affecting the behavior of tumor cells and the inflammatory microenvironment 32. In fact, the functions of NF-κB in cancer include evading apoptosis, promoting cell proliferation, angiogenesis and metastasis, and regulating cancer-related inflammation 32. Meanwhile, HIF-1α is a major transcriptional regulator involved in the hypoxia response 51, 52 and up-regulates a range of oncogenes to compensate for the hypoxia microenvironment and activates various angiogenic factors, leading to cancer recurrence and progression, thereby significantly reducing therapeutic efficacy 51, 52. NF-κB has been shown to regulate HIF-1α activation 51, 52. Additionally, VEGF, a downstream target of HIF-1α, is one of the most important angiogenic proteins 53. The NF-κB/HIF-1α/VEGF pathway plays an important role in stimulating tumor cell survival, proliferation, invasion, and metastasis by inhibiting apoptosis and regulating the immune system 13, 14. It has been confirmed that NF-κB, HIF-1α, and VEGF are significantly upregulated in cSCC tissues, and their expression is related to the metastasis and differentiation of cSCC 54-57. Blocking the NF-κB signaling pathway in cSCC cell lines down-regulates the expression of anti-apoptotic protein Bcl-2 and up-regulates the expression of the pro-apoptotic protein Bax, thus inducing apoptosis, and reducing cell activity 58. DHA was found to significantly inhibit activation of the NF-κB/HIF-1α/VEGF pathway in A431 cells, suggesting that DHA has a therapeutic effect in cSCC by inhibiting the NF-κB/HIF-1α/VEGF pathway.

Under normal conditions, autophagy can specifically remove endogenous activators of inflammasomes, thus limiting their activation 58. Moreover, autophagosomes can engulf inflammasome components such as AIM2, NLRP3, and ASC and transport them to lysosomes for degradation, thus directly regulating the assembly of inflammasomes 15. It has been confirmed that autophagy and NF-κB regulate each other. Specifically, autophagy regulates the NF-κB pathway primarily by degrading IKK components and NF-κΒ-inducing kinase 17.

We hypothesized that an upstream, or downstream, relationship exists between autophagy and the activation of the AIM2 inflammasome pathway and NF-κB/HIF-1α/VEGF pathway in A431 cells. We used an autophagy inhibitor (3-MA) or autophagy activator (rapamycin) to pretreat A431 cells before DHA treatment and found that DHA inhibits activation of the AIM2 inflammasome pathway and NF-κB/HIF-1α/VEGF pathway by promoting autophagy in A431 cells. Similarly, we treated A431 cells with ATG5 siRNA and DHA and obtained the same findings.

In conclusion, our study indicates that DHA inhibits the proliferation, invasiveness, and migration ability of A431 cSCC cells and promotes their apoptosis. Furthermore, DHA inhibits activation of the AIM2 inflammasome pathway and NF-κB/HIF-1α/VEGF pathway by promoting autophagy in A431 cells, thus exerting a therapeutic effect against cSCC. The study suggests that the induction of autophagy by DHA is mediated by inhibiting the mTOR pathway and promoting ROS production.
